# Intussusception in Infants1Read during the Twenty-third annual meeting of the American Association of Obstetricians and Gynecologists, held at Syracuse, September, 20-22, 1910.

**Published:** 1911-01

**Authors:** Herman E. Hayd

**Affiliations:** Buffalo, N.Y.


### BUFFALO MEDICAL JOURNAL

VOL. LXVI. JANUARY, 1911. No. 6

### ORIGINAL COMMUNICATIONS

### Intussusception in Infants[1]

By HERMAN E. HAYD, M.D., M.R.C.S., Eng.
Buffalo, N.Y.

[From *American Journal of Obstetrics*, January, 1911, by permission]

UPON looking over the transactions of our association since its inception, I find there have been but two papers published upon this important subject, and those were contributed by Dr. Henry Howitt, of Guelph, Ontario, in 1894 and 1898. Dr. Robert T. Morris, of New York, also at our Toronto meeting gave in 1894 a demonstration upon the production of intussusception in rabbits, by applying a little carbonate of soda to the exposed ileum. It seems strange that an association of active workers, many of them general surgeons connected with hospitals which have a large children's department, should have seen so little of this terrible malady and could have passed by unrecognised its sudden and dramatic symptomatology.

I believe, with other reporters in this field of work, that this condition is not uncommon but coming, as it does, in young infants who are so frequently the subjects of gastroenteritis and ileocolitis, the symptoms are often wrongly attributed to these diseases. However, when once seen and diagnosticated by the intelligent medical practitioner the picture is not easily forgotten, and so impressed is he with his first case that he very soon finds others. This fact has been demonstrated in the experience of many surgeons, as it was with Dr. Howitt who reported seven cases, three of which came from the same medical man. My case came to me from Dr. Nelson G. Russell, who diagnosticated the second one inside of six months, both being successfully operated.

It is interesting to study the surgical evolution of this subject. Intussusception in infants was recognised and accurately described even in ancient medical literature and, quoting from Clubbe's Monograph, "The clear and minute description of the mechanism and anatomy of intussusception, by John Hunter, could hardly be improved upon at the present day;" and yet it

1. Read during the Twenty-third annual meeting of the American Association of Obstetricians and Gynecologists, held at Syracuse, September, 20-22, 1910.

remained for Mr. A. A. Barker, in a paper in 1888, first to put the treatment of this subject on a rational basis.

Mr. Jonathan Hutchinson operated successfully a case in 1871, and yet in 1892, Mr. Hutchinson says, in an article published in the *Archives of Surgery,* London, Vol. IV, "If the patient be an infant, say under two years of age, it will be well to be content with repeated attempts by injection. The results of laparotomy in infants have been so almost invariably fatal that it is safer to trust to other measures." Yet, Clubbe, in his 1908 edition, Introductory Chapter on Intussusception, says that he personally has operated upon one hundred and twenty cases, with forty deaths, a mortality of 32.2 per cent. In the last twenty-four cases he had only three deaths, a mortality of 12.5 per cent; and I have recently seen a note in one of our medical journals that his last series was still more encouraging, owing to a better knowledge of the subject in his territory by the medical man, an earlier diagnosis, and an increased confidence in the brilliant achievements of modern surgery.

The most frequent spot for an intussusception is in the cecum, at the caput ceci, or in the ileum a few inches above the cecum; but it can take place in any portion of the intestinal tract, from the duodenum to the rectum. Many different classifications are made, but these divisions are of more pathological than clinical interest, since it is impossible to make a diagnosis of the exact type from any special signs or symptoms which ordinarily present themselves, or even upon inspection, until the mass is reduced.

The ileocecal and the ileocolic are the types which concern us most, since they occur most frequently in infancy, are very acute in their development, involve quite often a very large section of bowel, and are rapidly fatal, from the completeness of the bowel obstruction. The ileocecal occurs at the ileocecal valve and the valve is found to occupy the apex or lowest point of the intussusceptum; sometimes within a few hours the greater portion of the colon is involved and the apex of the tumor appears at the anus. The ileocolic resembles the ileocecal and is frequently confounded with it. It differs in having a few inches of the lower end of the ileum invaginated through the ileocecal valve, but in a few hours, owing to the tenesmic efforts induced, the cecum is forced into the colon and its after course exactly resembles that of the ileocecal. (Howitt.)

The colic and the enteric forms are not very acute and seldom produce complete obstruction, because they involve only a small portion of the bowel. There are many explanations offered as a cause for this interesting pathology. D'Arcy Power believes that it is mostly the result of anatomical conditions, and occurs when the colon is considerably larger than the ileum and when it is unduly moveable, owing to a long, lax mesentery. Others believe that the exciting factor is more physiological and is the result of some irritation of the bowel wall, that causes a spasm of its circular fibers, which reduces at that point the lumen of the gut, and then the small area is forced on by the powerful contractions of the longitudinal bands until an invagination is produced. Nothnagel says, "while the bowel is performing normal peristaltic movements, an annular and strictly local constriction of the bowels happens to occur. This constriction may be greater than normal and so pronounced that the limit of physiologic invagination is exceeded, and the first degree of pathologic intussusception develops."

Morris suggests, and upon these same grounds, that the toxalbumins and ptomaines consequent upon bowel fermentation and decomposition may suddenly irritate the circular muscle-tissue fibers and bring on a violent spasm, as was invariably produced experimentally upon the exposed ileum in twenty to forty seconds after the application of a few grains of carbonate of soda.

The diagnosis should be made easily, if one is careful to elicit the very definite history which always attends one of these sudden outbreaks. "Given, a baby who may have been previously quite well or had suffered from some bowel disturbance who, upon awakening from sleep, or after nursing, is suddenly seized with acute pain, screams and cries out, draws up its little legs, turns pale and vomits, and in a few hours passes blood, you can be morally certain of an intussusception." (Clubbe.)

The screaming does not last long and afterward the child whines and cries from the colicky pains. A reaction soon sets in, so that the baby looks very well, and the attendant and family can hardly believe in the possible existence of such a serious affliction. Soon after the first scream the child may have one or two natural movements, but in from two to ten hours, in 97 per cent. of all cases, blood will be passed per rectum.

Often, very early in the disease, one can feel a tumor or swelling high up across the median line or along the left of the navel, and if the finger is inserted in the rectum, blood and mucus will come away on it. No doubt, too much dependence has been placed upon the palpable existence of a tumor or sausage-shaped swelling, because in over 50 per cent. of the cases operated upon by Erdmann, no tumor could be felt; even when the abdomen was opened the mass lay so high up under the right and left costal cartilages, that the swelling could not be made out by any external palpation. If the case has advanced some hours, the tumor may be large, and then it sometimes can be felt through the rectum by the examining finger, or it may even point at the anus. Chloroform should always be given in making the examination if there be any question of doubt as to the diagnosis, because when the child cries and screams and makes its abdominal walls tense a proper examination cannot be made, and because, further, it is so important to completely establish a diagnosis at the first visit, that a necessarily fatal illness may be changed to a reasonably benign one by timely interference. It is surprising to see how young children, even in the early months of life, bear these surgical procedures, if not worn out by the suffering and the rapidly developing toxemia associated with this bowel obstruction.

The only condition that an acute intussusception can be confounded with is an acute colitis, in cases where the mesocolon is thickened, and feels very much like the intussusception tumor. The onset in both is acute, but in intussusception, after a few hours, the obstruction is complete and only foul smelling blood and slime is passed; whereas, in colitis, there are frequent motions of feces mixed with the blood and slime. The mass is also larger in colitis, but it can never be felt through the rectum. (Clubbe.)

Occasionally in an incomplete intussusception, as reported in two instances by Erdmann, where no blood was passed, and only a little mucus came away, the diagnosis would be very difficult and open to doubt. The treatment of this condition is surgical, and many of our best, most skilful, and most competent men never even attempt any other method of treatment. Mr. E. Owen of the children,s hospital, London, says: "I deem it nothing less than a calamity that physicians every now and then manage to chase back an intussuscepted piece of bowel by using an enema."

Mr. Clubbe, who has had such an unusual experience in the treatment of this disease, places the little patient on the operating table and prepares for an operation. Chloroform is given and an injection of about 16 ounces of olive oil or salt solution is made into the rectum, and even if every bit of the tumor disappears he sends the patient back to the ward; but if, in six hours, there is the slightest return of a swelling, or any of the symptoms of the disease, he operates at once. He says he has been successful in reducing only 14 out of 138 cases, 10.8 per cent. If in the hands of such a skilful diagnostician, who is also such an experienced and astute observer, the irrigation treatment effects a cure so seldom, then this treatment has fallen deservedly into ill favor.

Erdmann, who has also had a large experience, having operated upon 47 cases of all varieties and with all kinds of complications, condemns the irrigation as impractical and too uncertain. Evidently the risks of failure, with reduction by irrigation and insufflation, are so frequent and the possibilities of error in assuming a reduction to have taken place are so great, and the manifestly dangerous complications that result from a few hours' delay so imminent, that an operation becomes imperative just as soon as a diagnosis can be made. However, most surgeons agree that an injection should be given at the time of operation, not so much in the hope of effecting a complete reduction of the invagination, as with the object of reducing some of it, so that the subsequent manipulations when the abdomen is opened are more easily accomplished; and because, further, in many cases when the intussusceptum has engaged a large part of the transverse and even the descending colon, it is exceedingly difficult to reduce it without this preliminary irrigation, as the injection usually releases some of the mass from its intussuscipiens, or sheath.

An incision about two and a half inches in length is usually made on the side where the tumor is most prominent along the border of the rectus, or perhaps best in the median line, because it is easiest done and there is no bleeding, no vessels or nerves of importance being severed and, as a rule, the reduction is satisfactorily accomplished through this locality. If the tumor can be picked up and the invaginated part reduced, the bowels being retained in the abdominal cavity with gauze pads, this is always done; but sooner than waste any time by trying to hold the bowels back, fruitlessly grabbing a piece here and there, I think it is best at once to eviscerate, believing that the dangers of exposing the bowels is infinitely less than the added shock which results from the ineffectual, unnecessary, and awkward handling of the bowels within the peritoneal cavity. The tumor is lifted up and gentle pressure is made from below, at the apex of the swelling, between the thumb and fingers, when the enclosed mass usually slips back. No traction or at least only the slightest amount is ever made on the entering bowel. The bowel should then be stroked or gently stripped along its natural course for a few inches, to make sure that the reduction is complete and to help on the fecal current and its toxins.

If the case is not recent, the swelling of the inner cylinder is so great that reduction is often impossible, not from adhesions which Clubbe maintains seldom have time to form, but from the blood stasis and infiltration of the parts with serum and exudates. Here a resection of the part is often necessary, but unfortunately it is nearly always a fatal procedure. Sometimes the bowel here and there gives way during the efforts at reduction, and a number of superficial tears take place. These should be sewed up with fine catgut sutures. Often the bowels are so distended with gas that they cannot be pushed back into the belly cavity, hence occasionally it may be necessary to make a small incision into one of them to effect reduction. However, it is surprising to see how easily these distended coils can be returned, if the edges of the incision are hooked up by a tenaculum forceps, one on each side, and the little patient be lifted up from these two points, while the assistant, with soft gauze pads, gently pushes the bowels back. Salt solution and warm pads are used frequently throughout these manipulations, the omentum is pulled into position and, finally, the wound is closed with through and through silkworm sutures, a collodion dressing is applied, and then gauze and binder.

It is well before closing the abdomen to pass the fingers about in various directions to be certain that a second intussusception has not been overlooked, since multiple invaginations are not infrequent. A little morphia, 1|100 to 1|50 of a grain occasionally should be given, or small doses of paregoric if the stomach will bear it, to quiet pain and bowel peristalsis and to relieve shock; also such medicines and stimulation as may be expedient. When the vomiting has subsided a little albumin water can be taken and the child can be given the breast, or some suitable and properly diluted food.

The history of my case is as follows:

Margaret W., age three months, three weeks and three days, was operated on September 20, 1909, at 11.30 A. M., about 12 to 14 hours after the onset of the trouble. She was partly breast and partly bottle fed, and had been under the care of Dr. Nelson G. Russell, who had prescribed various combinations of food, because of the colicky condition always complained of. On the 19th of September, 1909, about 11 P. M., the mother telephoned him that the baby had awakened in terrible pain, and was screaming as if in agony. As the family lived some miles from the doctor's office, and as he had changed the food that day, he naturally thought the attack was like many previous ones, and he was content to prescribe a dose of castor oil and paragoric. On the following morning he made an early visit and at once diagnosticated intussusception.

I saw the baby with him about 11 o'clock; she was lying on the bed, awake, with her thumb in her mouth and evidently not in any great distress; but occasionally she winced and cried out as if suffering from colic. There was no fever, no distension, but upon palpating the left side of the abdomen high up above and to the left of the navel a distinct tumor could be felt; although none could be made out per rectum, yet blood and slime came away upon the examining finger. An injection of oil was given into the rectum, the abdomen gently kneaded and the little one was carried at once, by motor car, to the German Hospital. Chloroform was administered and then an incision was made along the left of the navel, about two and a half inches in length.

On opening the peritoneal cavity considerable clear fluid ran out. The tumor was at once seen and an attempt was made to reduce it within the belly cavity. In a very few minutes it was evident that this could not be effected, so the bowels were eviscerated, the swelling taken in the hand and pressure exerted from below up. It was some little time before it began to recede, and then only after the finger was slipped into the neck and a gentle stretching exerted on the tight ring. It proved to be of the ileocecal variety, commencing about three inches above the cecum and had engaged at least seven inches of the colon. The inner cylinder when released was of a dark maroon color, but the shining peritoneal luster was still present. The appendix was long, very dark and was in the mass, but it was not removed. The mesentery gave way at a few points in the reduction and a stitch or two of fine catgut was inserted at these spots; the bowels were then returned into the belly cavity with some effort, the omentum was pulled over them, and the wound was quickly closed with through and through silkworm sutures, collodion was applied and then gauze straps and binder.

The baby reacted beautifully and in sixteen hours had a fecal movement mixed with some blood and slime. Salt infusion, one ounce, was injected into the rectum every two hours, and about 1|100 of a grain of morphine was injected hypodermatically every third hour. She slept and rested nicely; was given distilled water frequently; then a little albumin water, and then diluted food in small quantities and often. She left the hospital on the ninth day. Dr. Russell assumed the medical management after the operation, and my nephew, Dr. Charles Gordon Heyd, assisted me in the surgical work.

In closing this subject, I may say that I am not surprised that many mistakes are made in diagnosis, not alone from carelessness, but really from want of knowledge, since the average medical graduate never heard a lecture on this important subject, and never saw a case during his student years. He is naturally content to classify every case, where blood and mucus is discharged from the bowels, as acute bloody dysentery, and signs a death certificate in 40 or 72 hours, attributing it to convulsions, or acute colitis, or acute dysentery. Surgeons must report their experiences with this rapidly fatal and yet easily curable affection, in the medical journals; also discuss the subject more freely before state and local societies, in the hope that a more general knowledge may be disseminated among the rank and file of the busy, practising doctors. They see these little sufferers first and most often, and if they make a quick diagnosis an early operation will cure 95 per cent. or more of patients suffering from this disastrous and rapidly fatal disease. Contrast the terrible mortality of appendicitis and extrauterine pregnancy of twenty-five years ago, under medical treatment, with the splendid results of today, due in no small measure to the work and teachings of this association; and we must not rest content until the same claim can be made for intussusception in infants.

493 Delaware Avenue.

